# Integrated diagnostics: the future of laboratory medicine?

**DOI:** 10.11613/BM.2020.010501

**Published:** 2019-12-15

**Authors:** Giuseppe Lippi, Mario Plebani

**Affiliations:** 1 Section of Clinical Biochemistry, University of Verona, Verona, Italy; 2 Department of Laboratory Medicine, University Hospital of Padova, Padova, Italy

**Keywords:** integrated diagnostics, laboratory medicine, pathology, radiology

## Abstract

The current scenario of *in vitro* and *in vivo* diagnostics can be summarized using the “silo metaphor”, where laboratory medicine, pathology and radiology are three conceptually separated diagnostic disciplines, which will increasingly share many comparable features. The substantial progresses in our understanding of biochemical-biological interplays that characterize many human diseases, coupled with extraordinary technical advances, are now generating important multidisciplinary convergences, leading the way to a new frontier, called integrated diagnostics. This new discipline, which is currently defined as convergence of imaging, pathology and laboratory tests with advanced information technology, has an enormous potential for revolutionizing diagnosis and therapeutic management of human diseases, including those causing the largest number of worldwide deaths (*i.e.* cardiovascular disease, cancer and infectious diseases). However, some important drawbacks should be overcome, mostly represented by insufficient information technology infrastructures, costs and enormous volume of different information that will be integrated and delivered. To overcome these hurdles, some specific strategies should be defined and implemented, such as planning major integration of exiting information systems or developing innovative ones, combining bioinformatics and imaging informatics, using health technology assessment for assessing cost and benefits, providing interpretative comments in integrated reports, developing and using expert systems and neural networks, overcoming cultural and political boundaries for generating multidisciplinary teams and integrated diagnostic algorithms.

## Introduction

**Figure 1:**
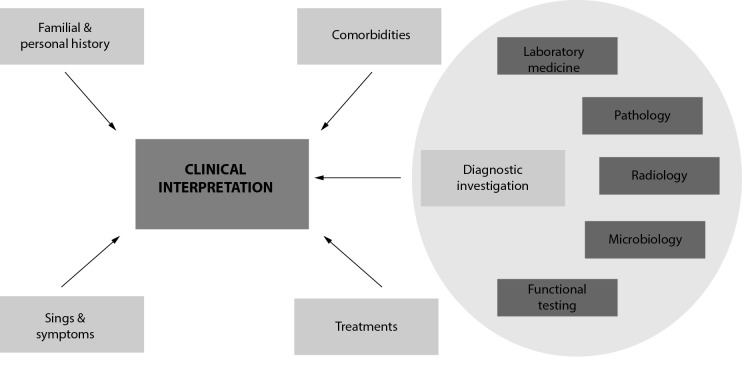
Clinical interpretation as a process based on multifaceted reasoning and many demographic, clinical and diagnostic domains.

Laboratory diagnostics is conventionally defined as a medical science aiming to generate useful clinical information by quantifying the concentration, composition or structure of many different analytes in different biological fluids (*1*). The everyday activity of laboratory services encompasses performing many different tests for generating qualitative, semi-quantitative or, most commonly, quantitative data. These numbers (alternatively referred to as “values”) can then be transformed into useful medical information by clinical interpretation, a process developing through experience, practice, knowledge and continuous critical analysis, which is actually based on a multifaceted reasoning where the different pieces (*i.e.* demographical variables, familial and personal history, signs and symptoms, results of diagnostic investigations, comorbidities, treatments) of the intricate puzzle (*i.e.* the patient) are combined (Figure 1) (*2*).

There is a long and still unresolved debate regarding how much of the decision-making process can be influenced by laboratory testing (*3*). It is now virtually unquestionable that the celebrated “70% claim” (*i.e.* “the clinical laboratory intervenes in 70% of clinical decision”) is not evidence-based, whereby this percentage may be consistently lower or higher depending on many variables such as the clinical setting, the case-mix, the human expertise and skill, and so forth (*4*, *5*). Two opposite but paradigmatic examples of how heterogeneous is the contribution of *in vitro* diagnostics to modern medicine are non-ST elevation myocardial infarction (NSTEMI), where the diagnosis can only be made with assessment of cardiac troponins, *versus* acute intestinal ischemia, where no single diagnostic biomarker achieves sufficient diagnostic accuracy for enabling to make an early and accurate diagnosis (*6*, *7*). Irrespective of clinical or environmental scenario, several lines of evidence now attest that the role of the so-called “integrated diagnostics”, defined as “convergence of imaging, pathology, and laboratory tests with advanced information technology (IT)”, will overwhelmingly emerge in the foreseeable future, allowing to make earlier and more accurate diagnoses, but also contributing to save a large amount of human and economical resources (Figure 1) (*8*-*10*). A better comprehension of several biological pathways, coupled with emerging technological advances, will foster a paradigm shift in the way diagnostics has been for long acknowledged, paving the way to a new model of healthcare where integration of many different data will be more rapid, efficient and straightforward, thus enormously amplifying the armamentarium that physicians could exploit to manage their patients (*11*).

**Figure 2:**
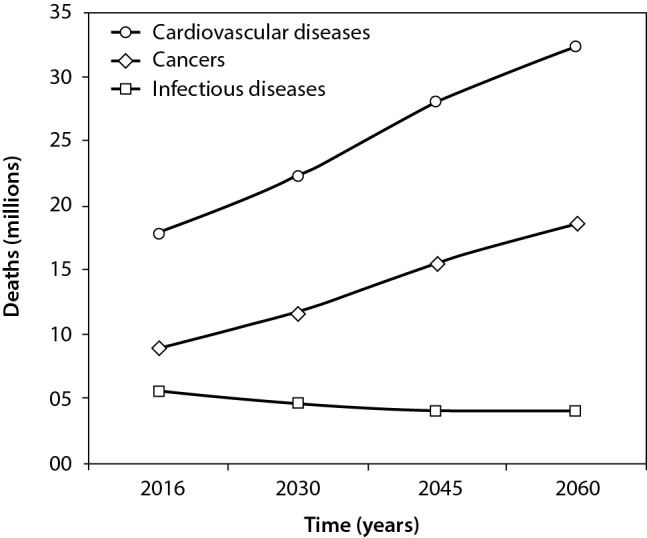
Current World Health Organization data and future trends of mortality for cardiovascular disease, cancer and infectious diseases (*12*).

According to the most recent statistics of the World Health Organization (WHO), the three worldwide leading causes of death are cardiovascular diseases (17.9 million deaths/year; 31.4% of all deaths), cancer (9.0 million deaths/year; 15.8% of all deaths) and infectious diseases (5.5 million deaths/year; 13.6% of all deaths) (*12*). Although the global mortality for infectious diseases is expected to decrease by approximately 30% by the year 2060, the number of deaths for both cardiovascular disease and cancer will exhibit a virtually linear increase in the next 40 years (Figure 2) (*12*, *13*). If diagnosis and treatment will not substantially improve, the mortality for these two conditions will nearly double by the year 2060. We will hence take these three foremost examples, which will continue to generate the largest clinical, societal and economic burden on humanity, for discussing the current scenario and the future perspectives of integrated diagnostics (Table 1).

**Table 1:** Current scenario and future perspectives of using integrated diagnostics for diagnosing the three leading causes of worldwide mortality

**Disease**	**Laboratory medicine**	**Pathology**	**Radiology**	**Other specific tests**
**Cardiovascular disease**				
**Acute myocardial infarction**	Cardiac troponins	-	Angiography, echocardiography, cardiac magnetic resonance imaging	Electrocardiography, stress testing
**Ischemic stroke**	Protein S100B	-	Non-contrast computed tomography, magnetic resonance imaging	-
**Venous thromboembolism**	D-dimer	-	Compression venous ultrasonography, computed tomographic pulmonary angiography or lung scintigraphy or magnetic resonance angiography	-
**Cancer**	Cancer biomarkers, liquid biopsy	Histopathology, molecular biology	Ultrasonography, computed tomography, magnetic resonance imaging, positron emitted tomography	-
**Infections/sepsis**	Sepsis biomarkers, lactate, blood culture, serology, molecular biology	-	Ultrasonography, radiography, computed tomography	-

### Integrated diagnostics in cardiovascular disease

The global burden of cardiovascular disease is mostly sustained by ischemic heart disease (IHD; 9.4 million deaths, 52.8% of total cardiovascular mortality), followed by stroke (5.8 million deaths, 32.4% of total cardiovascular mortality, the majority of which ischemic) and venous thromboembolism (VTE; 1.83 million deaths, 10.2% of total cardiovascular mortality) (*13*). In all these conditions, the role of integrated diagnostics has been clearly highlighted.

### Integrated diagnostics in acute myocardial infarction

According to the fourth universal definition of myocardial infarction, acute myocardial infarction (AMI) is diagnosed in patients with clinical evidence of acute cardiac ischemia, with a rise and/or fall of cardiac troponin values and with at least one measurement exceeding the 99^th^ percentile of the upper reference limit (URL) (*14*). This clear-cut definition poses laboratory diagnostics at the centre of the diagnostic reasoning, whereby patients with suspected AMI who do not display suggestive values of cardiac troponins will be immediately and accurately ruled out. On the other hand, however, an increased cardiac troponin value is not indicative of an ischemic injury, but can be frequently observed in patients with a kaleidoscope of non-ischemic cardiac and extra-cardiac pathologies (*6*). This clearly implies that cardiac troponins have an extraordinarily high negative predictive value (NPV) for AMI, up to 99%, but their positive predictive value (PPV) is often lower than 50%, thus requiring additional elements for value interpretation (*6*). Although the clinics is always an essential aspect to influence the clinical decision making, reliable evidence attests that some radiologic tests may provide a very important contribution to the diagnostic approach of patients with heart diseases.

Coronary angiography has represented for decades the mainstay for identifying and/or localizing restrictions or obstructions in heart vessels, thus enabling the accurate identification of culprit lesion(s), prompt establishment of appropriate therapeutic measures (*e.g.* coronary revascularization), as well as stratification of short- and long-term risk of major adverse cardiovascular event (MACE), including reinfarction and death (*15*). The combination of coronary angiography and cardiac troponin testing seems now virtually unavoidable for improving clinical outcomes and reducing healthcare costs (*16*).

More recently, the fourth universal definition of myocardial infarction has first introduced the concept that cardiac magnetic resonance imaging (MRI) may be used for more clearly defining the aetiology of myocardial injury, thus providing an adjunctive and valuable contribution to laboratory testing and electrocardiography (*14*). Interesting evidence has then been provided that this technique may be used for assessing the infarction area, alone or in combination with cardiac troponins (*17*). Magnetic resonance imaging seems especially useful for studying patients with cardiac troponin-positive symptoms and unobstructed coronaries, in whom an alternative diagnosis could be made (*e.g.* acute or chronic myocarditis, Tako-Tsubo cardiomyopathy, and so forth) (*18*).

Interesting evidence is also emerging on the incremental value of combining laboratory and radiology investigations for predicting all-cause and cardiovascular death in healthy people. A large meta-analysis including five studies and 34,028 healthy subjects concluded that those with positive coronary calcium score (*i.e.* number, areas and peak numbers of calcific lesions detectable with computed tomography (CT)), have a ~8-fold enhanced risk to die during a follow-up of 45 months compared to those with negative calcium score (odds ratio (OR), 8.43; 95% confidence interval (95%CI), 6.25 - 11.36; P < 0.001) (*19*). Nearly identical conclusions were published in another large meta-analysis, encompassing eight studies and 6521 type 2 diabetic patients, whereby subjects with positive calcium score had ~5 fold higher risk of death during a follow-up of 5 years compared to those with negative calcium score (relative risk (RR), 5.47; 95%CI, 2.59 - 11.53; P < 0.001) (*20*). Notably, a recent study published by Korley *et al*. found that the combination of coronary computed tomography angiography (CTA) for quantifying coronary artery calcium score with high-sensitivity cardiac troponin enables to identify with 100% NPV a subset of patients at low risk of MACE (*21*). The validity of this approach has been recently confirmed in another study, showing that the addition of high-sensitivity cardiac troponin tests to coronary calcium score significantly improves the classification of patients at low and high risk of obstructive coronary disease (net reclassification index, 0.062; 95%CI, 0.035 - 0.089) (*22*).

### Integrated diagnostics in stroke

According to the 2018 guidelines of the American Heart Association/American Stroke Association (AHA/ASA), the diagnosis of acute ischemic stroke (AIS) is made with non-contrast computed tomography (NCCT), whilst the use of diffusion-weighted MRI (DW-MRI) remains questionable and currently limited to patients with negative NCCT findings (*23*). Although the AHA/ASA 2018 guidelines do include any biomarker in the initial diagnostic approach of patients with suspected AIS, evidence has been published that some laboratory tests may provide a valuable contribution to the diagnostic reasoning, especially protein S100B, neuron-specific enolase (NSE), glial fibrillary acidic protein (GFAP), myelin basic protein (MBP), and D-dimer (*24*). Among these biomarkers, the most solid evidence has been garnered for protein S100B. A recent meta-analysis concluded that the circulating values of this protein could be efficiently used for differentiating patients with AIS from healthy controls (mean difference, 85.5 pg/mL; P < 0.001) (*25*). A previous critical review of the literature also highlighted that protein S100B values correlate with stroke infarct volume, severity and with functional outcome (*26*). Therefore, a solid way has been paved for evaluating the cost-benefit of a possible combination of S100B measurement and NCCT in routine diagnostics.

The paradigm of ischemic stroke management lies in the straightforward concept that “time is brain”, so that treatment shall be started as soon as possible, preferably within 3 hours according to the AHA/ASA guidelines (*23*, *27*). Universal agreement has been reached that thrombolytic therapy (*i.e.* alteplase) shall not be given to patients with AIS displaying platelet count < 100 x10^9^/L, international normalized ratio (INR) > 1.7 and activated partial thromboplastin time (APTT) > 40 s (*23*). Therefore, performance of these laboratory tests may be often necessary, especially when the suspicion of an underlying coagulopathy is particularly high. The AHA/ASA guidelines also recommend routine measurement of blood glucose in all patients with AIS (*23*). Persistent in-hospital hyperglycaemia during the first 24 hours following an AIS is associated with worse outcome, thus making it reasonable to treat hyperglycaemia for achieving glucose values between 7.8-10.0 mmol/L and also establishing strict monitoring for preventing the possible onset of hypoglycaemia (*23*).

### Integrated diagnostics in venous thromboembolism

The generic definition of venous thromboembolism (VTE) encompasses the combination of two distinct but often associated clinical entities, *i.e.* deep vein thrombosis (DVT) and pulmonary embolism (PE) (*28*). This condition represents perhaps the most paradigmatic example of how integrated diagnostics is necessary for making a timely and accurate diagnosis. Irrespective of the clinical guidelines that are used, the basic concept underneath is that a diagnosis of VTE necessitates a thoughtful integration of pre-test clinical probability (based on history taking, signs and symptoms), results of laboratory testing and diagnostic imaging (compression venous ultrasonography, computed tomographic pulmonary angiography or lung scintigraphy or magnetic resonance angiography) (*29*-*31*).

Although a vast array of biomarkers has been evaluated for diagnosing VTE, there is now universal agreement that D-dimer is the biochemical gold standard (*32*). Due to its extraordinarily high NPV, typically comprised between 97-99%, this test has been placed at the pinnacle of virtually all diagnostic algorithms, whereby a negative test result would allow to safely rule out an acute thrombotic episode with > 99% accuracy in low-risk patients (*29*-*32*). Likewise cardiac troponins for AMI, however, D-dimer is characterized by a very limited PPV (often < 50%), which would need additional elements for value interpretation (*32*). D-dimer values are consistently increased in a large number of relatively frequent physiological (*e.g.* older age, pregnancy) and pathological (*e.g.* infections, cancers) conditions, which can only be identified or excluded with diagnostic imaging (*33*). In this respect, integration of clinical, laboratory and radiologic findings by means of information (expert) software systems was proven to be a reliable strategy for improving diagnostic efficiency and resources usage in different healthcare settings (*34*-*36*).

The term “theranostics” (or “theragnostics”) is used to denote treatment strategies combining diagnostics with therapeutics (*37*). In brief, it combines a diagnostic investigation that allows detecting a certain pathology, with a therapeutic agent that is then contextually delivered at the exact site of disease. Among the various examples of ongoing theranostics projects, the most representative of which is indeed precision medicine in cancer therapy, thrombus-targeted fibrinolysis is emerging as a promising diagnostic and therapeutic option for patients with thrombotic disorders (*38*, *39*). Immunoconjugates or biocompatible nanoparticles have been used for precisely localizing blood clots and for delivering targeted thrombolysis, which would ultimately increase the anti-thrombotic effectiveness and decrease the risk of hemorrhagic events. The essential breakthrough of this diagnostic and therapeutic strategy is represented by the use of molecular imaging with different tracers (*i.e.* conventional anti-fibrin antibodies, fibrin beta chain antibodies, anti-D-dimer antibodies, cyclic fibrin-binding peptides), and different imaging modalities (optical techniques, MRI, positron emission tomography (PET), single photon emission computed tomography (SPECT)), which help constructing an ideal bridge between technologies used in laboratory medicine and radiology (*40*-*42*). Notably, a recent study showed that the use of high-resolution *in vivo* optical molecular imaging with near-infrared fluorescence (NIRF) fibrin-specific reporters permits to identify venous thrombi and dissect their vulnerability to fibrinolysis, thus amplifying the diagnostic accuracy and therapeutic effectiveness (*43*).

### Integrated diagnostics in cancer

Pathology has remained at the heart of cancer diagnostics for decades, since it has been the virtually unique branch of diagnostic medicine capable to identify malignant disease and defining the type of cancer, the stage and even the potential therapeutic vulnerability (*44*). The many progresses in our understanding of cancer biology (*e.g.* the discovery of genetic mutations and epigenetics determinants that drive cancer growth), coupled with notable technical advances, have considerably mutated the background and the role of pathologists in recent years, paving the way to development of relatively innovative diagnostics fields, namely molecular diagnostics and genomic profiling. Diagnostic imaging also plays an essential role in cancer care, since it participates to the initial diagnosis, and then contributes to surveillance, follow-up and therapeutic monitoring (*45*). The third pivotal diagnostic branch, laboratory medicine, is also deeply involved in cancer diagnostics. Its current contribution is minimal for the diagnosis, at least compared to that provided by pathology and radiology, since it is almost limited to assessment of a narrow number of biomarkers such as prostate specific antigen (PSA) for prostate cancer screening, human papilloma virus (HPV) molecular diagnostics for the screening of cervical cancer, or faecal occult blood test (FOBT) for colorectal cancer screening (*46*-*48*). The vast majority of other molecular or phenotypic biomarkers that can be measured in routine clinical laboratories are instead used for disease monitoring and for guiding prognostic and therapeutic decisions (*49*). Unlike other class of pathologies, integrated diagnostics is already a strong realism in cancer diagnostics, whereby almost each and every guideline or recommendation include a discrete number of laboratory, pathology and radiology investigations (*50*). Digital pathology and large-scale computational analysis are also strongly emerging, and preliminary data shows that their combination with “radiomics” (*i.e.* the extraction of a large number of features from radiographic images using data-characterization algorithms) will enable significant advances in diagnostic and prognostic accuracy (*51*).

A similar “molecular revolution” that has involved pathology, and only marginally radiology, is now deeply engaging laboratory medicine (*52*). The analysis of aberrant pathways at molecular level is no longer limited to tissue samples obtained with invasive procedures (*i.e.* biopsies, surgery), but can now be made also in blood and in other biological fluids, by means of a much less invasive venipuncture. The often misused or abused term “liquid biopsy” is precisely defined by the US National Cancer Institute (NCI) as “a test done on a sample of blood to look for cancer cells from a tumour that are circulating in the blood or for pieces of DNA from tumour cells that are in the blood” (*53*). According to the NCI, liquid biopsy can hence be employed for early cancer detection, for defining the most appropriate therapy, for monitoring therapeutic response, and for early identifying malignant recurrence. Future decision-making strategies should hence leverage tissue- and blood-based biomarkers, as well as advanced imaging technologies (*e.g.* MRI, scintigraphy, PET) (*54*). The resulting combination of blood signatures, digital pathology and radiomics is the most efficient approach for more accurately diagnosing, characterizing and treating cancer in the near future. Biologically-driven interventional radiology and theranostics hold great promises in the way a subset of cancer patients more likely to respond to targeted chemotherapies will be identified (*50*).

### Integrated diagnostics in infectious diseases

Sepsis is the leading cause of death in patients with infectious diseases, accounting for up to 270,000 deaths every year in the US (*55*). Respiratory infections, especially pneumonia, are the most frequent causes of systemic disease, and are responsible for the overall highest number of deaths (*56*).

There are at least two foremost reasons that support the need of an accurate and timely diagnosis of sepsis. The first, and the most obvious, is straightforwardly summarized in the “It’s About TIME” mantra of the Sepsis alliance, which highlights that the prognosis of this condition is essentially time-dependent (*55*). Rapid care, within the so-called “golden hours”, is hence crucial for preventing disease transition into irreversible illness. The second important aspect is that a timely identification of responsible pathogens would prevent misuse or overuse of antibiotics, thus limiting the risk of antibiotic resistance, which has been recognized by the WHO as a growing threat to global health (*57*).

There is now incontrovertible evidence that *in vitro* diagnostics plays an essential role in sepsis diagnostics, whereby it provides irreplaceable contributions to screening (measuring procalcitonin, presepsin, C reactive protein and lactate), diagnosis (*i.e.* serology, blood cultures, nucleic acid analysis), therapeutic monitoring (by longitudinal monitoring of procalcitonin), and establishing the prognosis (by means of procalcitonin or with the Sequential Organ Failure Assessment (SOFA) score, which includes platelet count in combination with creatinine and total bilirubin concentration) (*58*-*60*). Notably, the role of lactate is essential in patients with sepsis, since its concentration reflects tissue hypoperfusion and correlates with mortality (*61*).

The important role played by diagnostic imaging in sepsis patients has been emphasized by current guidelines for management of severe sepsis, whereby performance of imaging studies for confirming a potential source of infection is one of the three essential criteria that should be used for diagnosing sepsis along with clinical findings and results of microbiological testing (*61*). Although no specific radiologic signs may be present in sepsis patients, chest radiography is helpful for detecting pneumonia and ruling out other potential causes of pulmonary infiltrates (*e.g.* pulmonary hemorrhage, metastases, pleural effusions). Computed tomography scanning or MRI may also be used, since they are more sensitive than conventional radiographies for detecting a vast array of possible sources of infections in chest, abdomen and other bodily districts (*62*).

**Figure 3:**
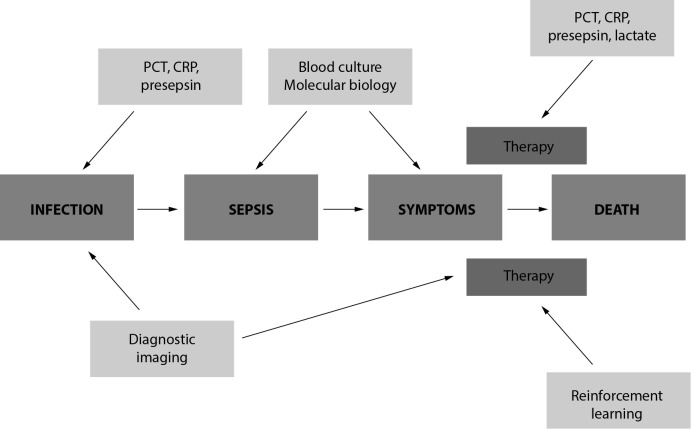
The many domains of an integrated diagnostic approach in patients with sepsis. PCT – procalcitonin. CRP – C-reactive protein.

Notably, the role of IT is also convincingly emerging in sepsis management. A recent study showed that promising results (*i.e.* lower mortality) have been obtained by implementing reinforcement learning for supporting sequential decision-making problem in patients with sepsis (*63*). As for the previous cases, therefore, a path towards integrated diagnostics is now unavoidable in sepsis care (Figure 3).

## Discussion

**Figure 4:**
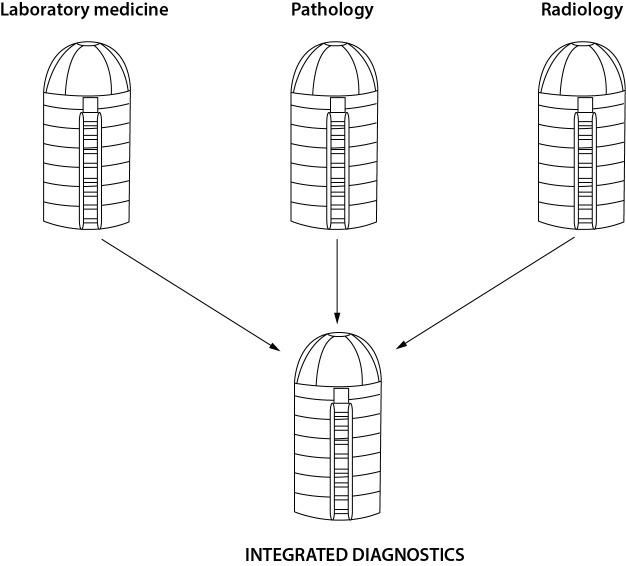
The “silo metaphor”, characterized by development of integrated diagnostics from convergence of laboratory medicine, pathology and radiology information.

The current scenario of *in vitro* and *in vivo* diagnostics has been effectively depicted by Lundström *et al.*, using the “silo metaphor”, where laboratory medicine, pathology and radiology are three conceptually separated disciplines sharing many comparable features, especially in terms of complex exploratory pathways (Figure 4) (*64*). Nevertheless, the considerable technological advancements and the extraordinary progresses occurred in our current understanding of the biochemical-biological interplay characterizing many human diseases are now generating considerable multidisciplinary convergences, leading the way to new frontiers in integrated diagnostics.

In the previous sections of this review, some paradigmatic examples have been discussed, corresponding to the three leading causes of worldwide deaths, to provide an overview on the current scenario and future perspectives of integrated diagnostics (Table 1). Cardiovascular disease, cancer and infectious disorders represent only a part of the many pathological conditions that may benefit from convergence and full integration of laboratory medicine, diagnostic imaging and pathology. Although it seems unavoidable that this process shall be further catalyzed and supported, there are some important obstacles that should be overcome, but there are also some possible solutions that can be identified (Table 2).

**Table 2:** Drawbacks and potential solution in integrated diagnostics

**Drawbacks**	**Potential solutions**
Infrastructure of information technology	Integrate exiting information systems Develop new integrated information systems Combine bioinformatics and imaging informatics
Costs	Health Technology Assessment
Enormous volume of different information	Include (increase) expert comments in integrated reports Develop and use expert systems and neural networks Overcome cultural and political boundaries Create multidisciplinary teams Introduce integrated diagnostic algorithms

The IT infrastructure can perhaps be seen as one of the biggest drawbacks in integrated diagnostics. The current laboratory, radiology, pathology and even hospital information systems have been constructed and developed independently, so that their connectivity is poor and functional integration is challenging, time-consuming and expensive (*65*). Although partial integration of data is occasionally feasible, especially within some hospital or “regional” information systems, a new culture should be developed, characterized by development of new software programs that will enable to collect, consolidate and integrate a large volume of different data within the same information system, producing a fully integrated electronic health report that will combine radiology, laboratory and pathology data, enabling a more detailed view of patient and care path (*66*). Convergence of bioinformatics and imaging informatics will then be necessary for combining biology, imaging, computer science, information engineering, mathematics and statistics, to help analyzing and interpreting a huge amount of biological information (*67*).

Unfortunately, cost will be another major limiting step in development of integrated informatics platforms. Overcoming this hurdle will not be easy, since public funding in healthcare is substantially declining everywhere around the world, due to the residual effect of an unprecedented economic crisis (*68*). The use of Health Technology Assessment (HTA) can be seen as a valuable solution for this problem, whereby the HTA definition *per se* encompasses a multi-professional and multidisciplinary assessment of cost-effectiveness of health technologies, based on active involvement of many different medical and diagnostic disciplines (*69*). Clear demonstration that an earlier and more accurate diagnosis will determine a return of initial investment for implementing integrated diagnostic platforms will lead the way to increased interest and funding.

The possible convergence of laboratory, pathology and imaging test results within the same medical report then implies that an enormous volume of different information will challenge the mind of healthcare professionals, especially of those who are directly in charge of the patient and will need to take the most appropriate medical actions, unravelling many intricacies. Reinforcement of clinical decision support through expert interpretation and counselling will become unavoidable (*70*). We should not forget that laboratory professionals act as managers of valuable clinical information and not as generators of raw numbers (*71*). In summary, laboratory professionals produce knowledge and the practice of laboratory stewardship, intended as clinicians’ guidance towards appropriate interpretation and use of laboratory information, is a central part of our routine activity (*72*, *73*). Artificial intelligence and machine learning may also be used for this scope, with development of expert systems or neural networks capable to integrate different information and assist decision-making abilities (*74*, *75*). All the available evidence, generated by heterogeneous diagnostic disciplines, shall then be combined into integrated diagnostic algorithms, which should carefully take into account advantages and the limitations of each test. This would inevitably require overcoming cultural and sometimes political boundaries among healthcare professionals, for establishing or reinforcing the cooperation between scientific societies of different medical diagnostic disciplines, throughout creation of collaborative interdisciplinary teams at local, national and, preferably, even at a supranational levels (*64*). An essential issue in this efforts to provide integrated and high quality information is represented by harmonization of clinical data. In particular, further efforts should be promoted to provide harmonization and standardization in laboratory medicine, not only in the analytical phase but also in all steps of the total testing process (*76*-*78*).

A final important issue concerns the education needed for using integrated diagnostics. Laboratorians, pathologists and radiologists have faced and eagerly won many technical and practical challenges that have revolutionized their work during the past decades (*37*). Nevertheless, becoming an expert in integrated diagnostics will probably be one of the hardest challenges ever since, because biological and technical background will need to be enormously magnified. Two different scenarios may be portrayed, the former directed towards creation of the figure of a “Radio-Patho-Laboratorian”, who will work in a revolutionized “diagnostic room” where, potently supported by IT and artificial intelligence, this new healthcare professional will integrate laboratory, pathology and radiology data for providing a final interpretative support to the clinicians. In the second scenario, instead, the daily work of laboratorian, pathologists and radiologists will remain almost unchanged, but an unified interface will be made available to clinicians, where patient data will be accessible in a more mineable form. We do not have a crystal ball, and we cannot predict the future. Neither we have a preference on either of these solutions. Only the future will tell which will be the dominant one.

In conclusion, integrated diagnostics can be considered one of the greatest opportunities for future healthcare, since it would permit to deliver more patient-centric care, obtain better outcomes and ultimately decrease cost over time. The chance to merge different diagnostic modalities within a single medical record will also boost the development of population-level databases, containing aggregated information on million of patients, and thus enabling to achieve a more holistic picture of many human diseases and developing more effective treatment. Additional efforts shall hence be made to foster the collaboration among different diagnostic disciplines, overcoming cultural, political and technical boundaries, for developing the diagnostic discipline of the future. Will integrated diagnostics contribute to put an end to laboratory medicine, at least as we now know it?
